# An ontology-based method for formalizing and encoding patient colonoscopy preparation using BPMN and OWL2 for automated tool development

**Published:** 2025-04-23

**Authors:** Muhammad Amith, Yue Yu, Yuheng Shi, Brooks Cash, Barani Mayilvaganan, Anirudh Babu, Yang Gong

**Affiliations:** 1 Department of Biostatistics and Data Science, School of Public and Population Health, University of Texas Medical Branch, Galveston, Texas, United States of America; 2 Department of Internal Medicine, John Sealy School of Medicine, University of Texas Medical Branch, Galveston, Texas, United States of America; 3 Department of Clinical and Health Informatics, McWilliams School of Biomedical Informatics, University of Texas Health Science Center at Houston, Houston, Texas, United States of America; 4 Department of Internal Medicine, McGovern School of Medicine, University of Texas Health Science Center at Houston, Houston, Texas, United States of America

**Keywords:** Process modeling, Knowledge engineering, Ontology, Knowledge graph, Semantic web, Consumer health informatics

## Abstract

Colonoscopy is a proven procedure for mitigating the incidence of colorectal cancers in late adulthood. Despite its effectiveness, colonoscopy preparation presents several challenges for patients, including issues with adherence and misunderstanding. Information and communication technology (ICT) tools may offer valuable support for modern patients. To enable such tools, computable knowledge bases are essential. In this paper, we describe the development of an ontology-based knowledge graph representing four laxative-based pre-colonoscopy procedures. We used the business process modeling and notation and OWL2 formalisms to manifest machine-readable artifacts that model processes of colonoscopy preparation. Future work will focus on integrating extended knowledge bases from biomedical ontologies and developing prototype ICT tools to support decision-making in colonoscopy preparation.

## 1. Introduction

Colorectal cancer is a malignancy that originates from polyps located in the rectum or colon, which may evolve into cancerous lesions over time.^1^ Common symptoms include bloody stool, unexplained weight loss, abdominal discomfort, and abnormal bowel habits.^2^ Risk factors for colorectal cancer include a family history of the disease, lifestyle choices (e.g., smoking, alcohol abuse, and diet), and genetic dispositions.^3,4^

From an epidemiological and population health perspective, colorectal cancer accounts for 10% of all cancer incidences.^5^ In 2024, it was reported that there were 152,810 new colorectal cancer cases in the United States, with 53,010 individuals dying from the disease.^5^ Worldwide, colorectal cancer is the third most common type of cancer and one of the leading causes of cancer-related mortality.^6^ Early diagnosis, along with consistent screening, significantly reduces mortality; when detected early, the survival rate is about 90%, underscoring the critical importance of timely screening and diagnosis.^7^

### 1.1. Overview of colonoscopy

Colonoscopy is a widely accepted and effective diagnostic procedure for the early detection of colorectal cancer. It involves examining the inner lining of the rectum and colon to identify precancerous polyps. During the procedure, a tubular camera (i.e., colonoscope) is inserted through the rectum to provide real-time video imaging of the colon’s interior. The procedure typically takes between 30 min and an hour and is recommended for adults aged 45 and older as a preventive measure.

Before undergoing a colonoscopy, patients must complete a bowel preparation procedure 1 or 2 days before the appointment. This preparation includes dietary restrictions and the ingestion of prescribed laxative mixtures to cleanse the bowel.^8^ Failure to adhere to the preparation protocol may lead to complications, including increased medical costs and greater discomfort during the procedure.

### 1.2. Information and communication technology (ICT) tools for colonoscopy preparation

ICT tools offer an opportunity to improve colonoscopy preparation for patients. These tools may include messaging reminders, instructional videos, interactive chat systems, and websites.^9–13^ A systematic review provides evidence that mobile applications could help address some of the challenges associated with colonoscopy preparation.^9^

Although many mobile and non-mobile consumer applications exist, they tend to suffer from unstandardized and inconsistent content. As a result, these tools are often underutilized and lack the impact necessary for real-world application. Researchers have also mentioned that many of these applications fail to provide standardized instructions for colonoscopy preparation.^9^ Similarly, printed materials – such as flyers and pamphlets – often vary in quality and presentation across different providers.

We propose that it is necessary to formalize and comprehensively describe the information and knowledge surrounding colonoscopy preparation. Once standardized and translated into a machine-readable format, this knowledge could serve as the foundation for innovative, interactive ICT tools designed to improve patient adherence and colonoscopy outcomes.

### 1.3. Process modeling in health care

Colonoscopy preparation involves multiple steps, resources, and variations depending on the specific method used. Essentially, these procedures constitute a defined process, often communicated to patients through instructional brochures. We argue that such planned processes represent essential information components when developing tools or analytics aimed at improving health outcomes from colonoscopies. Therefore, these planned process needs to be explicitly modeled and represented as machine-processable artifacts.

Process modeling refers to the structural representation of planned processes or workflows, with the aim of improving process efficiency and effectiveness. Many industries engage in process modeling, often using approaches inspired by the manufacturing industries. Healthcare processes are inherently complex^14,15^ and can potentially benefit from modeling techniques adopted in other industries. These techniques can contribute to a shared understanding of process flows, procedural specifications, clinical compliance, adherence, analytics, and more.^16^ In addition, process modeling in health care supports the interoperability of heterogeneous systems and workflows.^17–19^

Conventionally, health care processes are modeled from the provider’s perspective. Nonetheless, there is increasing interest in modeling workflows from the patient or health consumer view – a practice typically known as “patient journey modeling” or “patient journey mapping.”^20,21^ Applying a process modeling approach to colonoscopy preparation could facilitate the needed standardization of procedural communication and enable the integration of related processes, such as dietary restrictions and instructions for managing medications. Furthermore, if these processes can be translated into a machine-readable format, this would promote a process – first approach to the development of innovative ICT interventions.

### 1.4. Objective

To realize this vision for standardized, machine-readable procedures for colonoscopy preparation from the patient perspective, we propose the use of ontologies to construct and define machine-readable software artifacts using OWL2 (Version 2.0, World Wide Web Consortium [W3C]).^22,23^ We further propose employing business process modeling and notation (BPMN; Version: 2.0, Object Management Group, US)^24^ as a representational process modeling framework to draft and define these standard processes. OWL2 will then be used to translate the modeled procedures into a machine-readable format.

By encoding pre-colonoscopy procedures in a machine-readable format, we create opportunities to leverage technology-based solutions for improving patient readiness and compliance with colonoscopy appointments. The details of our approach are discussed in the next section.

Overall, we posited that OWL2 ontologies can effectively define core colonoscopy preparation procedures from the patient perspective, serving as a machine-readable knowledge base. The output of this work consists of four BPMN-represented knowledge graphs that describe colonoscopy preparation involving medication and laxatives. This work represents a first step toward developing a computable knowledge base for patient-facing ICT tools, as well as standardized, machine-readable models for medication-related procedures and processes.

## 2. Methods

We describe our process for developing an ontology-based representation of colonoscopy preparation. Starting with a collection of patient-directed documents (e.g., flyers and pamphlets), we created BPMN models to represent the patient preparation process. These were then translated into OWL2 artifact models for each variant of the colonoscopy preparation process. We evaluated the resulting BPMN-based ontology (BBO) knowledge graphs using a software reasoner in Protégé (Version: 5.6.5, Stanford Center for Biomedical Informatics Research, US) to validate the logical axioms, including logical satisfiability (accurate classification of data) and consistency (the absence of contradictory facts).

### 2.1. BPMN and BBO

**Figure 1.**
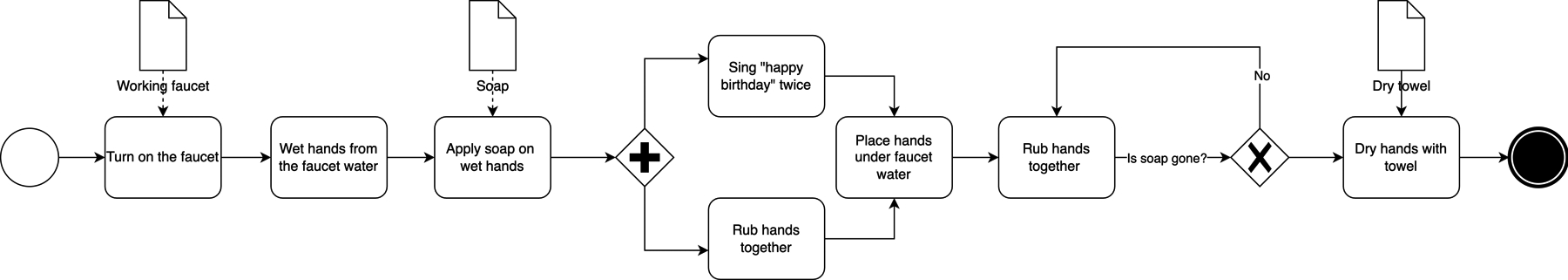
A simple business process modeling and notation diagram illustrating hand washing, adapted from a centers for disease control and prevention online flyer.^26^ The diagram uses rectangles to represent tasks, artifact symbols for resources, and diamonds to denote decision points or parallel processes.

BPMN is a graphical language used to describe business processes. Similar to standards such as Unified Modeling Language activity diagrams,^25^ BPMN leverages a set of notational elements that explicitly communicate complex activities and procedures. Some of these elements are widely adopted in systems and process modeling – for example, diamonds to represent decision points (“Gateways”), rectangles for tasks or activities, and directed lines to connect single action elements. Figure 1 displays a simple yet significant example of using BPMN to model the health-related process of hand washing as a means to mitigate virus transmission.^26^

Ontologies are representational artifacts that symbolically describe knowledge and information using controlled domain-specific terminologies.^27^ Essentially, they are formalized knowledge graphs in which semantic connections between terms form a network graph model. The building block of an ontology is an informational triple (i.e., subject > *predicate* > object), expressing a meaningful connection between two entities (the *subject* and the *object*) with a semantic connection (predicate). In this framework, linked entities may correspond to process models, where tasks and stages are semantically connected.

In the domain of artificial intelligence, ontologies enable machines to understand the contextual meaning of entities in the real world and to reason with that knowledge. In addition, since ontology-based models are machine-readable, they allow software systems to perform complex tasks and reasoning with the help of semantics. Furthermore, ontologies serve as schemas for knowledge graphs, ensuring the formalization of the structure.

General ontology development principles encourage the reuse of existing terminologies or ontologies to promote interoperability and facilitate validation.^27,28^ Accordingly, we sought to adopt a reusable BPMN ontology representation. After reviewing the availability of open-sourced BPMN ontologies, we selected the BBO 2.0,^29,30^ which is encoded as an OWL2 artifact and distributed under the GNU General Public License v3.0 (GPLv3).

For brevity, the core classes of this ontology include Process, Task, SequenceFlow, Gateway, Event (StartEvent and EndEvent), ConditionalExpression, InputOutputSpecification, and Resources. The Process class represents the entire process. Task denotes each atomic activity within the process. SequenceFlow serves as a sequential link between Task and Resource. Resources are entities that are either input to or output from processes or activities. InputOutputSpecification describes metadata relationships among Resource, Task, and Process. Gateway represents decision points or “forks” in the process, directing flow in different directions. ConditionalExpression is used in conjunction with Gateway to specify conditions for directing the flow of the process model.

### 2.2. Modeling the colonoscopy preparation process with BPMN

For this project, we focused on colonoscopy preparation using GolyTELY, MiraLAX, Sutab, and Pelenvu as representative use cases. According to WebMD, GolyTELY is a “laxative that works by drawing large amounts of water with the colon. This effect results in watery bowel movements.”^31^ MiraLAX is a powder-based laxative for treating constipation. “It works by holding water in the stool to soften the stool and increases the number of bowel movements.”^32^ Sutab consists of “tablets for oral use. an osmotic laxative indicated for cleansing of the colon in preparation for colonoscopy in adults.”^33^ Prevnu “is a prescription medication used by adults to clean the colon.. before a colonoscopy.”^34^ We collected and reviewed patient-directed instructions for each of these four colonoscopy laxatives.

For each procedure, we “normalized” the procedures by identifying shared activities and incorporating any unique but essential steps. This phase involved drafting and authoring BPMN representational models using draw. io.^35^ Co-authors, who are medical doctors specializing in gastroenterology, reviewed the BPMN models. The final output consisted of four BPMN process models representing the respective colonoscopy preparation procedures. These models served as a foundational draft for the knowledge graph development discussed in the next section.

### 2.3. Modeling the colonoscopy preparation process with BBO

We used BBO as a schema to structure and model instantiations for the four colonoscopy preparation procedures. For each BPMN model, we created a spreadsheet of the linked activities described in the process models. Each element (e.g., activity, process, annotations) in the BPMN process model was assigned a unique identifier. Each element (a source) was linked to another element (a target). For each source, we assigned a corresponding class concept from BBO and assigned a label that described the element (based on the annotation from the BPMN diagram).

We developed Java (version 11) software using the OWL API along with assorted software libraries (Apache POI v5.2,^36^ Google Guava v33,^37^ Apache Commons^38^). This software imports the spreadsheet information and converts the BPMN information into linked instances of BBO classes. These instances include class instantiations for activities, as well as assertions linking them, forming a linked structure modeling the process of colonoscopy preparation processes for patients. For conciseness, we describe the implementation in the following pseudo-code and snippets.

In Algorithm 1, how each BPMN process element is extracted from the tabular format. Essentially, each row *R* of the table *T* contains the corresponding values of the element identifier, the description of the action and task *x*, the associated BBO class *y*, and element’s linked target *g* (another BPMN element of the model). In line 3, these data points are stored in a node object *n*, which is then inserted in the BPMN node collection *N*^*c*^ (line 4). The link between the node and its target (line 5) is stored as a sequence object *s* (line 6) and added to the BPMN sequence collection *S*^*c*^ (line 6).

With the extracted BPMN process information for colonoscopy preparation, we encoded it using the BBO schema to generate the ontology-based knowledge graph *O* (Algorithm 2). Each extracted BPMN node object *n* is encoded as a knowledge graph instance *i* (line 2). After encoding each BPMN node, the referred BPMN elements *ri* of the sequence object *s* (line 5) are used to encode the link between encoded node instances *l* (line 6), forming the graph structure between BPMN elements in knowledge graph representation.

Finally, the knowledge graph (created in memory) is exported as a file artifact in.owl format. Listing 1 presents a simplified Java code snippet that demonstrates the use of the OWL API to perform this task.

## 3. Results

The outputs of our work include BPMN representations for four colonoscopy preparation procedures for patients (Figures S1–S4) – one each for MiraLAX, GolyTEYL, Sutab, and Plenvu. These models were reviewed by two physician co-authors with expertise in endoscopy (BC and BM). While the four protocols share a common set of tasks, they differ in specific instructions based on the prescribed laxatives. For example, GolyTEYL requires 2 h of consumption, while MiraLAX must be taken simultaneously with Gatorade. In addition to the BPMN diagrams, each process model was also captured in a corresponding tabular version with metadata, which served as the input for generating OWL2 representations.

Using an automated software program we developed, we generate OWL2 knowledge graph representations for each of the four BPMN-modeled processes, structured according to the BBO ontology. Table 1 presents an example of the tabular information derived from the BPMN process models. Table 2 summarizes the statistics for each of the four generated knowledge graphs – Golytely_KG.owl (Golytly preparation), MiraLAXGatorade_KG.owl (MiraLAX and Gatorade preparation), Plenvu_KG.owl (Plenvu preparation), and Sutab_KG.owl (Sutab preparation). On average, the graphs contained 374 generated instance data and 908 instantiated triples. All knowledge graphs are publicly available in our GitHub repository (https://github.com/ProfTuan/BPMN-Knowledge-Graph-Rendering-Engine/tree/main/examples/Colonoscopy%20Preparation%20Process).

**Algorithm 1:** Extracting BPMN element information from tabular resource

**Input:** *T* ⇒ {*R*^n^} is table resource T that contains a set of rows *R. N*^c^ and *S*^c^ are a collection of BPMN nodes and sequences, respectively.	
**1:**	**for** each *r* in *R* **do**
**2:**	*r={i, x, y, g}*
**3:**	*n*←*r*
**4:**	*Nc=Nc* ∪ *{n}*
**5:**	*s ← {i, g}*
**6:**	*Sc=Sc* ∪ *{s}*
**7:**	**end for**

Abbreviation: BPMN: Business process modeling and notation.

**Algorithm 2:** Encoding of the BPMN elements to knowledge graph instantiations

**Input:** *N*^c^ is a collection of BPMN nodes, S^c^ is a collection of BPMN sequences	
**Output:** *O*→{*i*_*n*_, *l*_*n*_} is ontology-based knowledge graph *O* that contains a set of instances *i* and linked instances l	
**1:**	**for** each node *n* in *N*^*c*^ **do**
**2:**	$i_{n}^{n}\leftarrow E(n)$
**3:**	**end for**
**4:**	**for** each sequence s in Sc **do**
**5:**	$ref(s)\to (ri_{n}^{n},ri_{n}^{n})$
**6:**	$l_{n}^{n}\leftarrow L(ri_{n}^{n},ri_{n}^{n})$
**7:**	end for

Abbreviation: BPMN: Business process modeling and notation.

**Listing 1:** Export phase for BPMN translation to a knowledge graph

**1:**	OWLOntologyManager manager;
**2:**	OWLOntology ontology;
**3:**	…
**4:**	Manager.saveOntology (ontology, ….);

Abbreviation: BPMN: Business process modeling and notation.

Ontologies and knowledge graphs that leverage the semantic structure of an ontology are logically represented using formal description logic provided by OWL2.^39,40^ Each colonoscopy knowledge graph was validated using the FacT++ software reasoned,^41^ a high-performance reasoner for checking logical consistency and satisfiability. We developed software to automatically import each knowledge graph from our GitHub repository and tested it with the reasoner. Our evaluation software code is also available on GitHub.^42^ The reasoner flags inconsistencies or unsatisfiable axioms through diagnostic messages. All BBO-based knowledge graphs passed the consistency and satisfiability checks.

**Figure 2.**
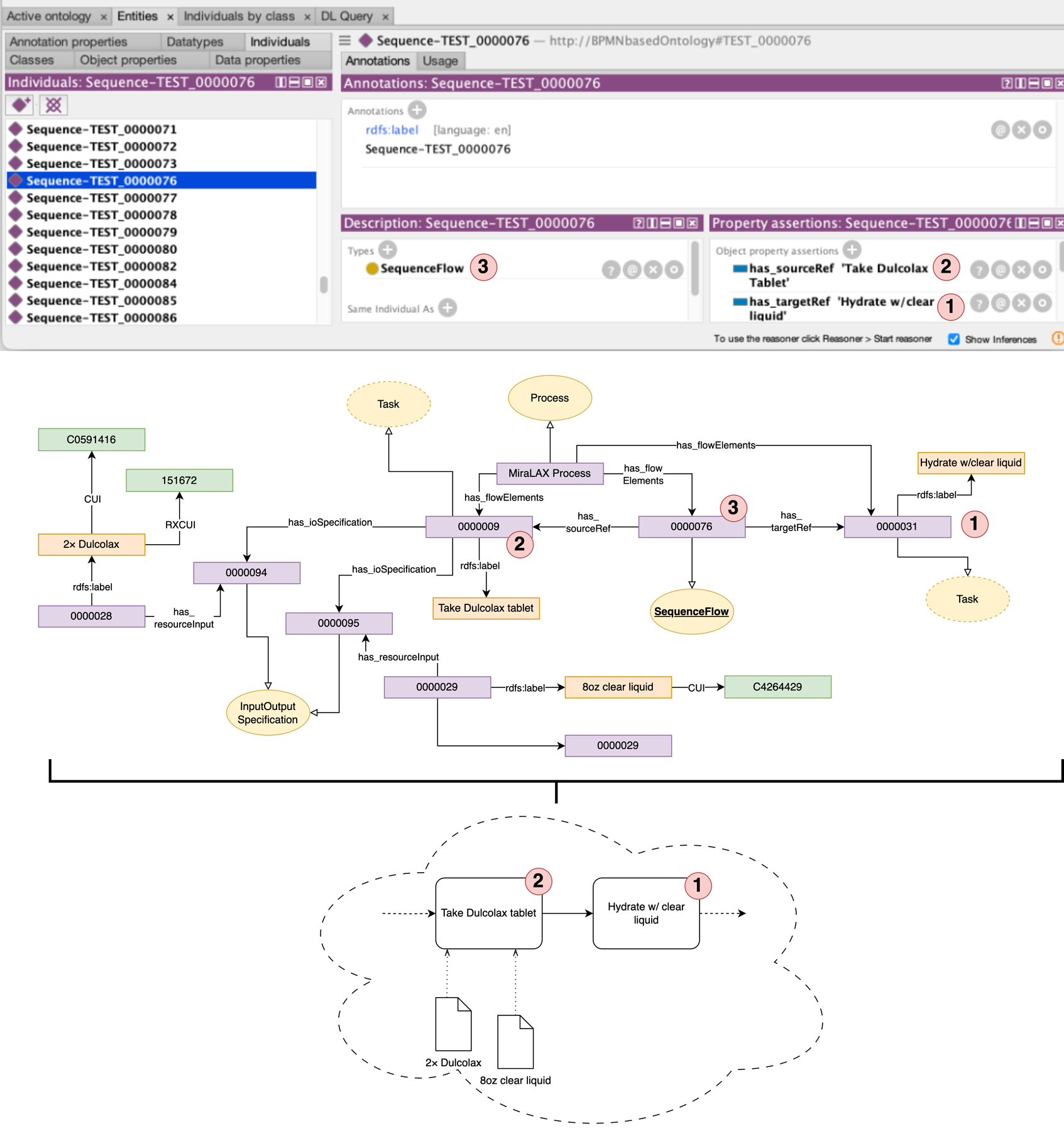
Ontological representation of a sequence flow in one colonoscopy preparation process model using business process modeling and notation, specifically the intake of clear liquid after consuming two Dulcolax tablets. Purple nodes represent instance-level data, yellow denotes classes, orange represents annotation labels, and green indicates external linked resources.

The Java software code and example ontology models are available in our GitHub repository under the GNU General Public License v3.0 open-source license (https://github.com/ProfTuan/BPMN-Knowledge-Graph-Rendering-Engine). The software can potentially be extended to generate ontology models for other colonoscopy preparation processes not covered in this study. Figure 2 displays the MiraLAX colonoscopy preparation process as an ontology in Protégé,^43^ illustrating an ontological sequence where hydration with clear liquid follows the administration of Dulcolax tablets. Each sequence described in our BPMN process models is denoted by a SequenceFlow instance (e.g., 0000076 in Figure 2), which links two Task instances. Each instance entity (Tasks in Figure 2) is assigned a unique seven-digit identifier, in alignment with Open Biomedical Ontology Foundry standards.^44^ Specific entities such as the tasks and resources are annotated with human-readable labels using rdfs: label (e.g., 0000028>rdfs: label>“2× Dulcolax”).

The knowledge graph also supports linking to external resources, such as standard controlled terminologies from SNOMED-CT and RXNorm (highlighted in green in Figure 2), enabling future extensions to related sub-processes in diet, nutrition, and medication adherence. Finally, every process element is associated with the main process entity (MiraLAX Process > has_flowElements > {0000009, 0000076, 0000031}).

## 4. Discussion

Process modeling is the practice of representing and standardizing workflows and all associated elements within a given domain. In general, process modeling holds significant potential in health care – for communicating health-related processes, reducing ambiguity through standardization, supporting decision-making, and enabling applications that facilitate analytical functions and automation.^45–48^ Graphical process modeling approaches,^49^ such as BPMN, enhance accessibility and promote widespread adoption. This motivated our decision to use BPMN as the foundational formalism for describing healthcare processes. Furthermore, relatively few studies have attempted to represent BPMN using ontologies.^30,50^

BPMN has become a well-known tool for process modeling due to its standardized visual notation, which provides a relatively intuitive and reliable means of communicating procedural information. However, unlike other industries (such as manufacturing), the healthcare sector has been slower to adopt BPMN, despite some reported use cases in the literature.^51^ A key contribution of this work is that we demonstrate a practical application of BPMN within the healthcare domain, specifically from the patient perspective. Furthermore, by providing machine-readable representations of these BPMN models, our work has the potential to realize some of the theoretical benefits of process modeling – particularly automation, a growing focus within health informatics.^52–55^

By leveraging BPMN and BBO to model and define colonoscopy preparation processes involving laxative medications, our work has resulted in a computable knowledge base encoded in OWL2. This contribution yields several benefits for clinical care, including the standardization of procedural knowledge and the development of an extensible, machine-readable representation of colonoscopy preparation procedures. The combined use of BPMN and OWL2 – two symbolic modeling languages – enabled us to formally and consistently define the workflow of colonoscopy preparation. BPMN provides a consistent language for describing processes that are relatively understandable and applicable across a wide range of subject matter experts and disciplines. However, we do not envision using BPMN as a communication format for patients and consumers; rather, we regard it as a professional standard or blueprint for the ontology department. OWL2 offers a semantic standard to translate the knowledge and information from BPMN-based processes into computable representations (with the assistance of BBO). Alongside OWL2 representations, an area we have yet to discuss is the affordance to extend our work. Given the wide availability of open-sourced biomedical ontologies, our representational models could be further enriched with knowledge bases developed by various community-driven, consensus-based efforts.

### 4.1. Limitations and future directions

While BBO provides an expressive representation of BPMN 2.0, we foresee the need to further extend its ontological representation for colonoscopy preparation procedures. First, there is a need to integrate auxiliary health information, as our current focus has been limited to the core procedural steps. The underlying knowledge graphs are relatively straightforward and simplified for patient-facing use. For example, we need to incorporate precautionary information, such as contraindicated medications or warnings about specific foods. This opens the opportunity to utilize existing open-sourced ontologies, such as those provided by Open Biomedical Ontology Foundry,^56^ and the National Center for Biomedical Ontology BioPortal,^57^ or to explore the development of new controlled vocabularies to fill potential information gaps.

Furthermore, the BPMN ontology we utilized appears to be designed primarily for industrial or general-purpose domains. Certain classes within the ontology, such as Resources, represent generic input and output materials. Consequently, we see a future direction in extending or developing a health care–specific adaptation of this ontology. For instance, the Resource class could be further subclassed to include material entities commonly found in clinical environments, such as medical devices and pharmaceuticals. In line with this, we are interested in reusing domain-specific controlled vocabularies from open-sourced ontologies. Examples include drug ontologies, food interaction ontologies (e.g., FIDEO),^58^ the Human Phenotype Ontology,^59^ and others relevant to medication adherence, patient safety, and clinical context.

One of the main drivers for this work is the development of ontology models that can be integrated into software applications to support colonoscopy patients through ICT tools. One such application is interactive conversational agents – particularly those that utilize ontologies as a knowledge base. Previous efforts have demonstrated the use of dialog interaction modeled as an ontology,^60^ which was then employed to drive counseling through an automated dialog engine for the human papillomavirus vaccine.^61,62^ We envision extending this approach using our ontology – designed to model patient-centered colonoscopy preparation procedures – as a complementary knowledge base alongside a dialog interaction ontology for counseling conversations on pre-colonoscopy preparations.^63,64^

## 5. Conclusion

We developed OWL2 representations for four major patient-centric colonoscopy preparation processes. These representations are based on BPMN and a conceptual-level ontology for BPMN—BBO 2.0. Our initial work, including Java software code for automatically generating the four ontology representations, is publicly available on GitHub (https://github.com/ProfTuan/BPMN-Knowledge-Graph-Rendering-Engine) and is released under the GPLv3 open-source license. Our future work will focus on expanding these models to include precautionary information and other relevant health-related data through the reuse of formal biomedical ontologies. In addition, we intend to develop complementary knowledge bases to support integration with future ICT tools aimed at improving patient education, readiness, and decision-making.

## Supplementary Material

Supplementary File

## Figures and Tables

**Table 1.** Example of the tabular information from the BPMN process models

ID	ACTION	Type	TARGET
AD1_22	Clock at 8PM	http://BPMNbasedOntology#TimerEvent	AD1_23
AD1_24	2×Dulcolax	http://BPMNbasedOntology#Resource	AD1_23
AD1_25	8oz Clear Liquid	http://BPMNbasedOntology#Resource	AD1_23
AD1_23	Take Dulcolax tablet	http://BPMNbasedOntology#Task	AD1_26
AD1_26	Hydrate w/clear liquid	http://BPMNbasedOntology#Task	AD1_27

Abbreviation: BPMN: Business process modeling and notation.

**Table 2.** Statistics for the generated knowledge graphs

Colonoscopy preparation knowledge graph	Instances	Triples
MiraLAX Gatorade Preparation	367	890
Golytly Preparation	375	914
Plenvu Preparation	367	877
Sutab Preparation	387	951

## Data Availability

All resources are available at our GitHub repository. Knowledge graphs: https://tinyurl.com/kg-colonscopy-prep Machine-readable BPMN models: https://tinyurl.com/bpmn-colonscopy-prep Source code (released under GPLv3 license): https://github.com/ProfTuan/BPMN-Knowledge-Graph-Rendering-Engine
