# Supplementary material for: An ontology-based method for formalizing and encoding patient colonoscopy preparation using BPMN and OWL2 for automated tool development
Source: J Clin Inform. Author manuscript; Available in PMC 2025 Sep 26. (PMC12461748)
Supplement: Supplementary File [file NIHMS2089131-supplement-Supplementary_File.pdf]

ACCSCIENCE PUBLISHING

Journal of Clinical Informatics

ORIGINAL RESEARCH ARTICLE

An ontology-based method for formalizing and encoding patient colonoscopy preparation using BPMN and OWL2 for automated tool development

Supplementary File

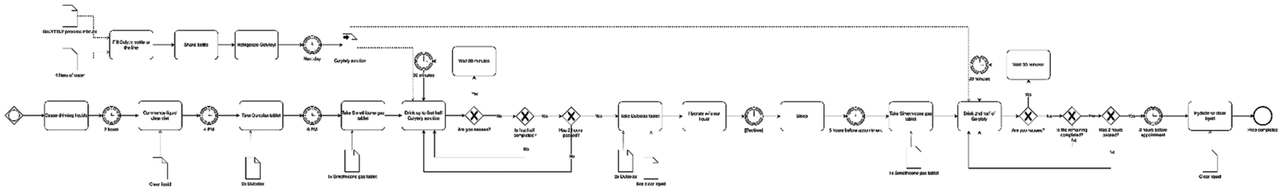

Figure S1. Business process modeling and notation model representation for colonoscopy preparation with GolyTELY

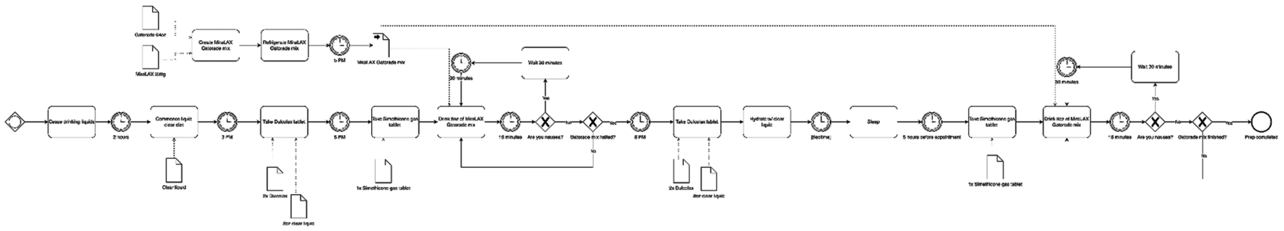

Figure S2. Business process modeling and notation model representation for colonoscopy preparation with MiraLAX

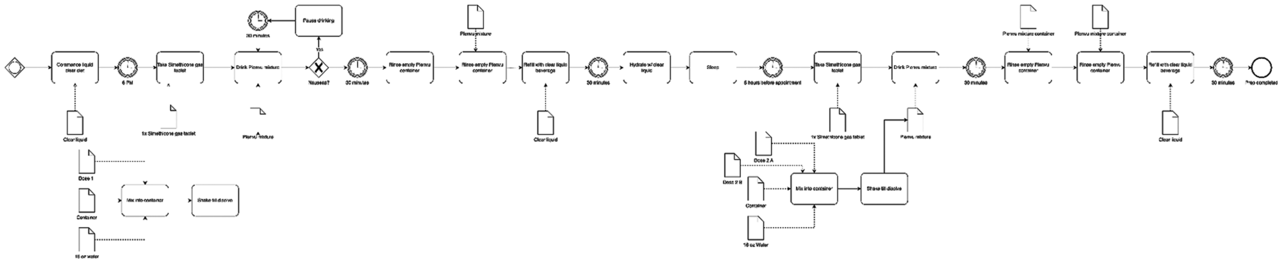

Figure S3. Business process modeling and notation model representation for colonoscopy preparation with Plenvu

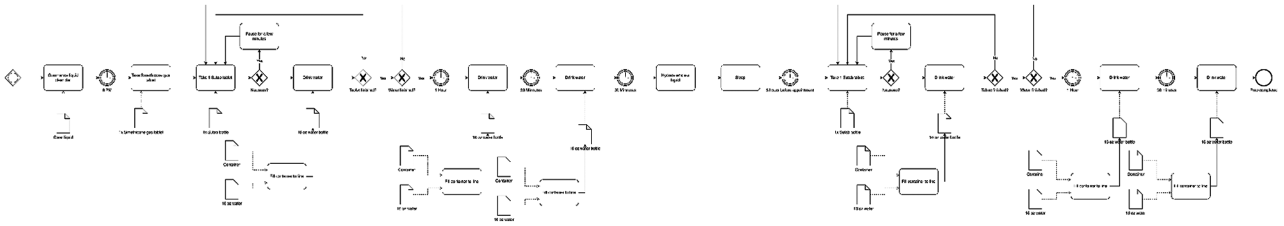

Figure S4. Business process modeling and notation model representation for colonoscopy preparation with Sutab
